# A Rare Case of Hashimoto’s Encephalopathy With Mosaic Turner Syndrome

**DOI:** 10.7759/cureus.28215

**Published:** 2022-08-20

**Authors:** Vijaya Chelikani, Deepti N Rao, Shravya Balmuri, Abdul K Arida

**Affiliations:** 1 Department of Internal Medicine, McLaren Flint/Michigan State University, Flint, USA

**Keywords:** encephalopathy, turner, autoimmune encephalopathy, hashimotos thyroiditis, hashimoto’s, turner mosaicism, hashimoto’s encephalopathy

## Abstract

Mosaicism in Turner syndrome (TS) is a 20%-30% occurrence, with 45, X plus at least another cell line. The haploinsufficiency of the X chromosome is usually responsible for the higher risk of autoimmunity in TS, exhibiting mainly as thyroiditis, type 1 diabetes, etc. Though Hashimoto’s thyroiditis is commonly seen in patients with TS, the concurrence of encephalopathy in these patients is significantly rare and has not been reported. We present a case of a young female with mosaic TS who presented with altered mental status. The initial workup was negative for stroke and pulmonary embolism and cerebrospinal fluid (CSF) analysis did not show any infectious etiology. Thyroid peroxidase (TPO) antibodies (Abs) and thyroglobulin Abs were elevated. As the patient’s mental status deteriorated, there was a concern for Hashimoto’s encephalopathy (HE), hence the patient was started on high-dose IV steroids. Within 24 hours, the patient responded to the IV steroids and an improvement in mentation was noted. HE is a rare immune-mediated disorder, characterized by impaired brain function. The onset of which can be rapid or slowly developing over the course of many years but responds effectively to steroids. Turner syndrome is associated with a high incidence of autoimmune disorders, thus in the setting of a negative workup for more obvious causes, HE should be a consideration when encountered in a clinical scenario.

## Introduction

Brain dysfunction resulting from global insults or focal lesions, that alters brain structure or function, is described as encephalopathy. The causes are manifold and can be difficult to pinpoint. One of the rare ones is Hashimoto's encephalopathy (HE), an autoimmune illness affecting approximately two in 100,000 [1]. Patients present with a wide variety of symptoms, ranging from mild confusion to seizures and coma. It may be associated with autoimmune thyroiditis and can only be diagnosed when toxic, metabolic, and infectious causes have been ruled out [2]. Neuro-imaging such as brain MRI is generally normal. Even though the pathogenesis and etiology are not fully understood, patients often respond well to corticosteroids. Other modes of treatment include intravenous immune globulin and plasma exchange. Several studies report an increased frequency of autoimmunity in Turner syndrome (TS) patients possibly due to a convoluted interaction between genetic and ecological factors [3]. To the best of our knowledge, there are no case studies of HE reported in TS patients. In this case report, we talk about a 33-year-old female with a past medical history of mosaic TS and hypothyroidism, who was diagnosed with HE and treated with steroids successfully.

## Case presentation

**Figure 1:**
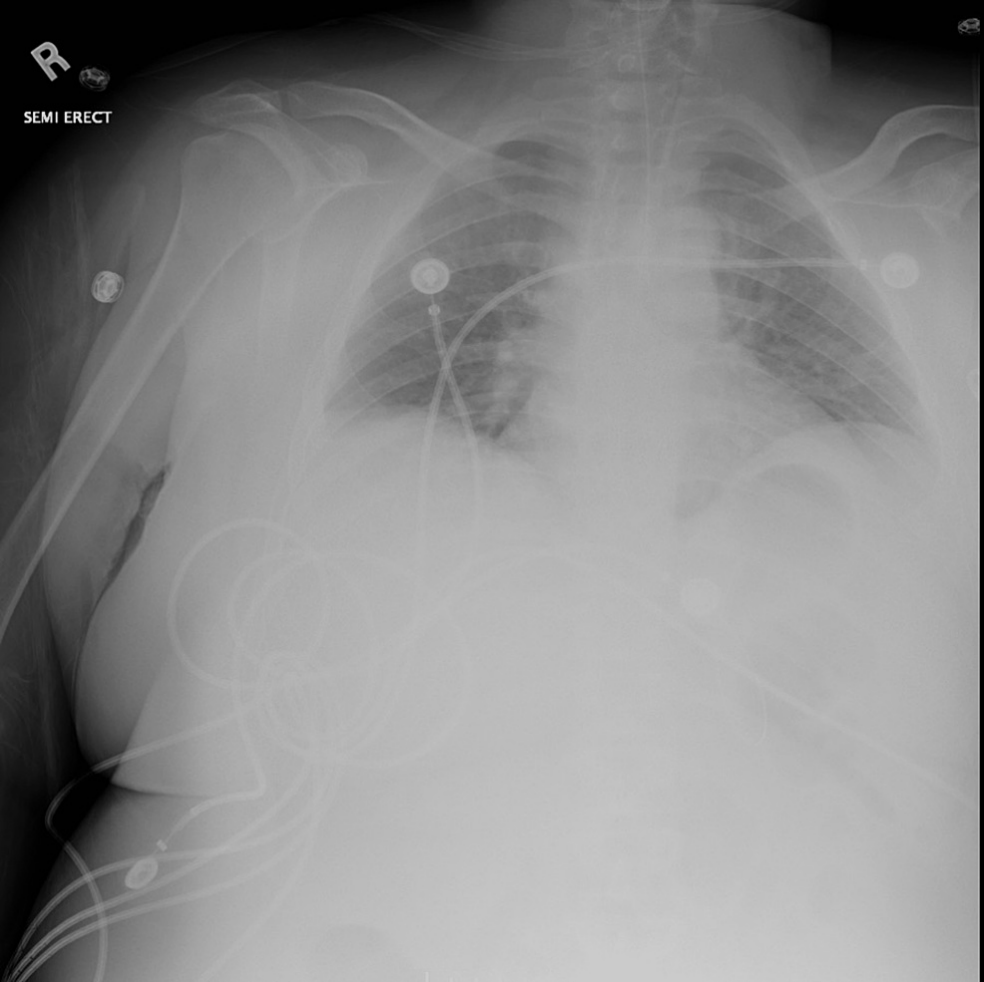
Chest x-ray

The patient is a 33-year-old Caucasian female with a past medical history significant for mosaic TS, cerebrovascular accident (CVA) 10 years prior with no residual deficits, type 2 diabetes, hypertension, and hypothyroidism who presented with acute altered mental status. At baseline, the patient had normal mental status. On presentation, according to her father, she was found with her face down on the bathroom floor along with fecal incontinence. Emergency medical services were called and the patient was brought into the emergency department (ED). On arrival at the ED, a stroke alert was called and imaging was done. CT head and CT angiography of the brain and neck were all negative. Imaging was significant for previously demonstrated patchy ground glass opacities on chest x-ray as shown in Figure 1, in view of her recent recovery from a bout of COVID-19 infection.

**Figure 2:**
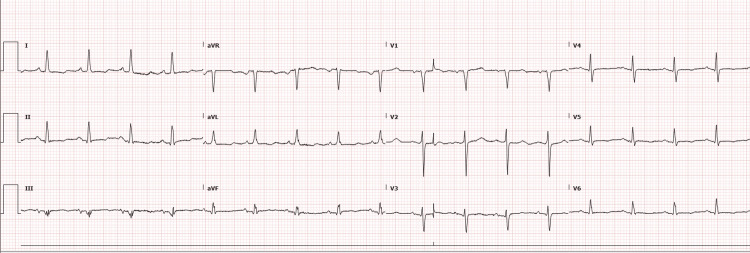
Electrocardiogram on admission

At the time of evaluation, the patient was aphasic, non-verbal, and moving all her extremities. Physical examination was significant for confusion, left eye herpetic lesions, upper extremity stiffness, expressive aphasia, and hyporeflexia with negative meningeal signs. Vitals were significant for hypertension and tachycardia, 173/116 mm Hg and 115 beats per minute, respectively. EKG, as shown in Figure 2, was in normal sinus rhythm. Labs were significant for troponinemia (0.06 ng/mL, 0.96 ng/mL) and she was started on an IV heparin drip along with other acute coronary syndrome (ACS) protocols. Initial differentials included stroke, seizure, meningitis, encephalitis, and other metabolic causes. Other significant labs were mildly elevated creatinine, D-dimer of 1.22, the thyroid-stimulating hormone of 33.5 mIU/L with normal free thyroxine (T4).

Supportive fluids and broad-spectrum antibiotics along with IV acyclovir in view of possible sepsis with meningitis were initiated. The patient was also started on IV levetiracetam 500 mg twice daily and IV levothyroxine in view of her altered mental status. The following day, an MRI brain was done which was negative for any acute process. EEG showed diffuse slowing along with mild asymmetry between both hemispheres, concerning for left hemispheric seizure. The dose of levetiracetam was increased to 1 g twice daily but there were no signs of any clinical improvement in the patient. Lumbar puncture was done and CSF analysis, as shown in Table 1, showed normal cell count and was negative for cryptococcal antigen, herpes simplex virus (HSV), and varicella-zoster virus (VZV) along with negative culture. The antinuclear antibody (ANA) screen was also negative. 

**Table 1:** CSF analysis CSF: cerebrospinal fluid; WBC: white blood cell count; RBC: red blood cell count; HSV: herpes simplex virus; VZV: varicella zoster virus; VDRL: venereal disease research laboratory test

CSF Test	Result	Normal Result
CSF color	Colorless	Colorless
CSF xanthochromia	Negative	Negative
CSF WBC	1	0-5
CSF RBC	0	0
Paraneoplastic antibody CSF	Negative	Negative
Cryptococcal antigen	Negative	Negative
HSV type 1 PCR	Not detected	Not detected
HSV type 2 PCR	Not detected	Not detected
VZV PCR	Not detected	Not detected
VDRL	Non-reactive	Non-reactive
Myelin basic protein	<2	2-4

Further workup revealed positive anti-peroxidase antibody and anti-thyroglobulin raising concern for HE. The patient was then started on a trial of high-dose steroids which led to significant improvement over 24 hours, in terms of alertness and intermittent cooperation with her parents. She completed a course of IV Solumedrol 250 mg every six hours for five days and was gradually weaned off it. There was a considerable improvement on day 5 of steroids and the patient was transferred to the inpatient rehabilitation unit for improving her functional status.

## Discussion

HE is a rare disorder, initially mentioned by Brian et al., in 1966 in a 49-year-old man with hypothyroidism who presented with features of encephalopathy, including intermittent confusion. The prevalence has been estimated to be 2.1/100,000, but there may be many other undiagnosed cases of lesser severity [1]. HE, like autoimmune thyroid disease, is more common in women and has been reported in all age groups around the world [3,4].

Due to its varied presentation, it has been further classified into two subtypes - a vasculitic type, characterized by multiple stroke-like episodes, and a diffuse progressive type, characterized by dementia and psychiatric symptoms [5]. In a study by Laurent et al. where 250 cases were reviewed, HE had various clinical presentations, from progressive cognitive impairment to psychiatric symptoms and coma [6]. Although there is no clear evidence, memory problems seem to be a common finding. Seizures (66%), myoclonus (38%), neuropsychiatric symptoms (36%), and stroke-like symptoms (27%) are also noted. EEG findings are not specific to any particular etiology. The most commonly noted abnormality is a slow wave background, associated with any type of encephalopathy, which was also seen in our patient and indicates the severity of her clinical status. The clinical presentation is subdued due to the non-specific symptoms which makes it very hard to diagnose [7,8].

According to the diagnostic criteria outlined by Castillo et al. and Chong et al., the diagnosis of HE requires fulfillment of the following (1) encephalopathy manifested by cognitive impairment and one or more of the following: neuropsychiatric features (e.g., hallucinations, delusions, or paranoia), myoclonus, generalized tonic-clonic or partial seizures, or focal neurologic deficits; (2) presence of serum thyroid antibody (TPO or microsomal); (3) euthyroid or hypothyroid status (4) no evidence in blood, urine, or CSF analysis of an infectious, toxic, metabolic, or neoplastic process; (5) no serologic evidence of the neuronal voltage-gated calcium channel, voltage-gated potassium channel, or other currently recognized paraneoplastic autoantibodies to indicate another diagnosis; (6) no findings on neuro-imaging studies indicating vascular, neoplastic, or other structural lesions to explain the encephalopathy; and (7) complete or near-complete return to the patient's neurologic baseline status following corticosteroid treatment [9,10]. All the above-mentioned criteria were present in our patient. 

In the study of 250 cases by Laurent et al., only 32% of the patients had known thyroid disease and most had normal TSH levels so it is important to check antithyroid antibodies periodically in a patient when the cause cannot be elucidated [6]. In a retrospective study by Castillo et al., in 20 patients who met the inclusion criteria, the levels of thyroid antibodies varied from case to case. All patients exhibited neuro-cognitive symptoms, but antibody levels did not correlate with the intensity of the clinical deficits indicating that the presence of antibodies and not the titer should be considered in making the diagnosis. One-third of the patients in the study by Castillo had evidence of multiple autoimmune disorders and it is a long-known fact that in patients with autoimmune thyroid disease, multiple other autoimmune disorders exist together [10]. Our patient with TS has a greater predisposition to autoimmune diseases, paving way to the possibility of a likely association between the two that has not been much studied. 

The risk of autoimmune diseases in patients with TS is approximately twice as high as in the general female population [3]. The genetic propensity to autoimmunity in patients with TS has been ascribed to X-chromosome haploinsufficiency, maternal origin of the X chromosome, excessive production of pro-inflammatory cytokines, decrease in anti-inflammatory cytokines or hypogonadism [3]. X chromosomes can be progressively lost leading to a subsequent haploinsufficiency of some X-linked genes, causing havoc on the functioning of the immune system. The mechanism is explained by the fact that auto-reactive T cells and other immune-related cells, are not tolerant to self-antigens encoded by one of the two X chromosomes, thus stimulating an autoimmune response [11,12]. HE is considered as an inflammatory autoimmune disorder due to its steroid sensitive nature but the actual etiology and pathogenesis have not been explained. Our literature review revealed several other possible etiologies, including hypothyroidism itself, humoral antibodies, antigen-antibody complexes, vasculitis, intrathecal thyroid antibodies, and global cerebral hypo-perfusion [13].

Treatment with steroids showed benefit in the majority of the cases. However, there was no added benefit in using intravenous steroids compared to oral prednisone. The timeline for response to steroids varied from a couple of days to a few weeks, with some showing a considerable improvement after the first few doses similar to what we witnessed in our patient [13]. The treatment protocols followed currently are high dose steroids in the range of 1-2 mg/kg of prednisone. Intravenous immune globulin and plasma exchange can also be effective as initial treatment or when there is no significant response to steroids [14]. There is some evidence that azathioprine may be helpful, usually when combined with corticosteroids. Other treatments that were tried include cyclophosphamide and methotrexate, with varying results [14].

It is a diagnosis of exclusion with diagnostic criteria that encompasses neurological or psychiatric symptoms, positive anti-thyroid antibodies, exclusion of other possible causes, and in most cases, an apparent clinical response to steroids or other immunosuppressants [14,15]. In our case, the acute onset of symptoms of altered mental status with normal imaging and non-specific labs, ruling out other obvious etiologies and the previous history of hypothyroidism and TS did shed light on the possibility of HE. However, the presence of thyroid auto-antibodies and the clinical response to steroids helped in clinching the diagnosis.

## Conclusions

HE still poses a diagnostic challenge due to its heterogeneous presentation and no well-defined diagnostic criteria. As mentioned in our case above, it is deemed to be a diagnosis of exclusion and should be considered in the differential diagnosis, after metabolic, infectious, and toxic causes have been ruled out, especially in a patient with Turner, which is associated with a high incidence of autoimmune disorders.

## References

[1] Pinedo-Torres I, Paz-Ibarra JL (2018). Current knowledge on Hashimoto's encephalopathy: a literature review. Medwave.

[2] Zhou JY, Xu B, Lopes J, Blamoun J, Li L (2017). Hashimoto encephalopathy: literature review. Acta Neurol Scand.

[3] De Sanctis V, Khater D (2019). Autoimmune diseases in Turner syndrome: an overview. Acta Biomed.

[4] Montagna G, Imperiali M, Agazzi P, D'Aurizio F, Tozzoli R, Feldt-Rasmussen U, Giovanella L (2016). Hashimoto's encephalopathy: a rare proteiform disorder. Autoimmun Rev.

[5] Churilov LP, Sobolevskaia PA, Stroev YI (2019). Thyroid gland and brain: enigma of Hashimoto's encephalopathy. Best Pract Res Clin Endocrinol Metab.

[6] Laurent C, Capron J, Quillerou B, Thomas G, Alamowitch S, Fain O, Mekinian A (2016). Steroid-responsive encephalopathy associated with autoimmune thyroiditis (SREAT): characteristics, treatment and outcome in 251 cases from the literature. Autoimmun Rev.

[7] Ramalho J, Castillo M (2011). Hashimoto's encephalopathy. Radiol Case Rep.

[8] Tomkins M, Cavalcoli F, Stanley E, O'Rourke K, Murphy S, Lynch T, Tamagno G (2018). Autonomic alterations as a clinical manifestation of encephalopathy associated with autoimmune thyroid disease. Endocr J.

[9] Chong JY, Rowland LP, Utiger RD (2003). Hashimoto encephalopathy: syndrome or myth?. Arch Neurol.

[10] Castillo P, Woodruff B, Caselli R (2006). Steroid-responsive encephalopathy associated with autoimmune thyroiditis. Arch Neurol.

[11] Bianchi I, Lleo A, Gershwin ME, Invernizzi P (2012). The X chromosome and immune associated genes. J Autoimmun.

[12] Larizza D, Calcaterra V, Martinetti M (2009). Autoimmune stigmata in Turner syndrome: when lacks an X chromosome. J Autoimmun.

[13] Marshall GA, Doyle JJ (2006). Long-term treatment of Hashimoto's encephalopathy. J Neuropsychiatry Clin Neurosci.

[14] Olmez I, Moses H, Sriram S, Kirshner H, Lagrange AH, Pawate S (2013). Diagnostic and therapeutic aspects of Hashimoto's encephalopathy. J Neurol Sci.

[15] Mocellin R, Walterfang M, Velakoulis D (2007). Hashimoto's encephalopathy: epidemiology, pathogenesis and management. CNS Drugs.

